# The Potential Role of Growth Hormone on the Endometrium in Assisted Reproductive Technology

**DOI:** 10.3389/fendo.2020.00049

**Published:** 2020-02-14

**Authors:** Fen-Ting Liu, Ze Wu, Jie Yan, Robert J. Norman, Rong Li

**Affiliations:** ^1^Ministry of Education Key Laboratory of Assisted Reproduction, Beijing Key Laboratory of Reproductive Endocrinology and Assisted Reproductive Technology, Center of Reproductive Medicine, Peking University Third Hospital, Beijing, China; ^2^Department of Reproductive Medicine, The First People's Hospital of Yunnan Province, Kunming, China; ^3^Robinson Research Institute and Fertility SA, University of Adelaide, Adelaide, SA, Australia

**Keywords:** growth hormone, endometrial receptivity, mechanisms, risk, ART

## Abstract

Growth hormone (GH) has been considered as an adjuvant treatment in human assisted reproductive technology (ART) for several years. Its action was largely attributed to an improvement of ovarian function and less emphasis was paid to its role in the uterus. However, there is increasing evidence that GH and its receptors are expressed and have actions in the endometrium and may play an important role in modifying endometrial receptivity. Thus, in this review, we firstly describe the existence of GH receptors in endometrium and then summarize the effects of GH on the endometrium in clinical situations and the underlying mechanisms of GH in the regulation of endometrial receptivity. Finally, we briefly review the potential risks of GH in ART and consider rationalized use of GH treatment in ART.

## Introduction

Growth hormone (GH), also called as somatotropin, is a protein of 191 amino acids with a three-dimensional structure that interacts with its receptor (1). The synthesis and secretion of GH is dynamically regulated by growth-hormone releasing hormone and somatostatin, which are both produced by the hypothalamus (2). In recent decades, GH has been considered to regulate many physiological functions including growth, metabolism, and reproduction (3, 4). Its therapeutic use in reproduction has been growing but remains controversial for its efficacy (4, 5). It has been used in assisted reproductive technologies (ART) in humans with an emphasis on potentially improved oocyte quality and pregnancy rates (6, 7). Little attention has been paid to the endometrium, despite the documented presence of GH receptors in this tissue (8). Our review of the literature indicates that study and application of GH for modulating the endometrium has been overlooked in understanding normal endometrial function in ART and reproduction generally. The endometrium undergoes changes via integrated interactions between the different uterine cell types and various growth factors and hormones (9). Several methods can be used to evaluate receptivity of the endometrium, including ultrasound, histology and molecular biomarkers (9, 10). Recent evidence has indicated that GH supplements may modulate endometrial receptivity and improve pregnancy outcomes in ART (10–13). Among women undergoing *in vitro* fertilization (IVF) treated with GH, changes of endometrial thickness (EMT) and endometrial perfusion have been described by ultrasound evaluation (11–14). The alteration of several biological markers of endometrial receptivity has also been detected with adjuvant administration of GH in animal models and cell-line studies (15–18). While short-term use of GH would not be expected to have problems currently, it could potentially have side-effects on other diseases, especially active cancer and metabolic diseases (19).

We describe the data on evidence of GH action and its receptor in the endometrium. We then mainly focus on the current evidence for the influence of GH on endometrial receptivity. Finally, we look at the potential risks of GH in co-treatment in ART.

## Methods

A comprehensive search of the literature available in the PubMed, Web of Science, Embase, and CNKI was conducted using the following keywords, MeSH terms and phrases in combination with one another; “growth hormone,” “somatotropin,” “endometrium/uterine receptivity,” “endometrial thickness,” “endometrium perfusion,” “endometrium cancer,” “disease,” “metabolism,” “side effect/adverse event” “uterus/endometrium,” “growth hormone knockout,” “infertility,” “reproduction” through July 2019. Both human studies and animal data were used.

## GH and Its Related Receptor in Endometrium

GH mediates its functions by binding to the GH receptor (GHR) (2). GHRs are most abundant in the liver (20), but also have been found in the reproductive system. GHRs have been reported in human granulosa cells and GH co-treatment in women receiving ART could regulate the expression of GHRs to improve pregnancy outcomes (7). The uterus also appears to be a site of both GH and GHR expression (2). GH has been detected in the cytoplasm of proliferating uterine epithelium cells in dogs (21) and also in human endometrial glandular cells during the mid and late luteal phases and in decidual tissue cells throughout pregnancy (22). GHRs can be found in uterine cells from various species including the mouse where localization of GHR mRNA in the endometrium, glands, stroma and myometrium have been described (23). GHR mRNA was also detected in the uterine epithelium, glands, vessels and placenta from bovine species (24) with biomolecular expressions including GHR and insulin-like growth factor-I (IGF-I) demonstrated in the uterus of dairy cows (25). In the pig, mRNA analyses demonstrated a high level of expression for endometrial somatotropin receptors (STR) (26). In women, GHR mRNA has been detected in the nuclei and cytoplasm of both human myometrial and leiomyoma cells (8). All these findings indicate a potential role for GH on the endometrium.

## Clinical Evidence of GH on Endometrial Receptivity

Endometrial thickness (EMT) and uterine perfusion are important clinical indicators of endometrial receptivity in ultrasound studies (10). It has been suggested that ultrasonographic parameters including EMT and uterine perfusion can predict implantation potential in infertile patients undergoing embryo transfer (27). Although this is controversial (28), recent studies suggest a positive relationship between EMT and pregnancy outcome (29–32). Patients with positive pregnancy outcomes following IVF treatment had thicker endometrium readings on the day of hCG administration compared with those where a pregnancy did not result (29). The thicker the endometrium evaluated on the day of human chorionic gonadotropin administration, the higher the pregnancy rates reported following IVF (30, 31). EMT can also be measured on the day of oocyte retrieval and have been alleged to predict the endometrial receptivity during fresh IVF cycles (32). In general, EMT should exceed 8 mm as the threshold of endometrial receptivity in fresh embryo transfer cycles (33), although other studies suggest 10 mm of EMT may be better for a more stable implantation of embryos and minimization of pregnancy losses (34). Hence, increasing endometrial thickness and uterine perfusion might be beneficial goals for improving endometrial receptivity.

Two reports of women with panhypopituitarism causing either primary or secondary infertility who were treated with GH and gonadotropins are illustrative of the potential role for GH in fertility promotion (35, 36). After GH treatment, an improvement in their response to gonadotrophin stimulation was demonstrated with an acceptable endometrial growth and successful pregnancies ensued (35, 36). Standard infertile patients also show different endometrial changes and different pregnancy outcomes after adjuvant GH treatment (Table 1). For infertile women classified as poor responders, GH treatment has been promoted for improving the chances of pregnancy and live birth outcomes. Although no significant increases in implantation or clinical pregnancy rates are consistently demonstrated, there appears to be an increase of retrieved oocyte numbers and EMT (39, 42). A large scale retrospective clinical trial of infertile women classified as normal responders also had an increase in endometrial thickness in the older group (age ≥ 35 years) utilizing GH treatment and an improvement of implantation rate (IR) and clinical pregnancy rate (CPR) was claimed in the GH treatment group across all ages (45). An effect on weight-related infertility has also been seen with a significant improvement of EMT, IR and CPR in a group of infertile women who were overweight and obese (BMI ≥ 24 kg/m^2^) (43, 45).

**Table 1:** The clinical evidence of GH on endometrial receptivity to improve pregnancy outcomes in ART.

**Years**	**Study design**	**Objectives**	**Samples**	**Programme**	**Intervention**	**Outcomes**	**Effects**	**References**
2007	Retrospective study	Infertile women with GH deficiency	20	IVF/ICSI	12 IU GH every third day, starting on the day of gonadotropin stimulation, till the administration of hCG	EMT FR	(–) ↑	(37)
2011	Prospective study	Infertile women with RIF	55	IVF-ET	4IU GH daily until the day of hCG administration	EMT IR/CPR	↑ ↑	(38)
2012	Randomized prospective study	Infertile women with poor responder	40	IVF-ET	4 IU GH from day 21 of previous cycle until the day of hCG injection	EMT IR/CPR Retrieved oocytes Obtained embryos FR	(–) (–) ↑ ↑ ↑	(39)
2013	Prospective study	Infertile women with endometrial dysplasia	32	FET	4IU GH daily until the day of hCG administration	EMT Trilaminar pattern Uterine perfusion CPR	↑ ↑ ↑ (–)	(40)
2015	Retrospective study	Infertile women with thin endometrium (EMT <7 mm)	35	FET	4IU GH daily, starting from the 3–5th day of menstrual cycle, until the day of progesterone administration	EMT CPR	↑ ↑	(41)
2016	Parallel randomized, open-label study	Infertile women with poor responder	68	IVF/ICSI	2.5 mg(7.5IU) GH daily, starting on day 6 of hMG stimulation until the day of hCG triggering	EMT IR/CPR	↑ (–)	(42)
2016	Prospective study	Infertile women with overweight/obesity	33	IVF-ET	4.5 IU GH daily, starting from the day of hMG administration till the day of hCG	EMT IR/CPR	↑ ↑	(43)
			34	IVF-ET	4.5 IU GH every alternate day, starting from the day of hMG administration till the day of hCG	EMT IR/CPR	↑ ↑	(43)
2016	Prospective study	Infertile women with thin endometrium (EMT <8 mm)	5	FET	4-5 times of GH intrauterine perfusion (6IU/0.5 ml 0.9% saline) on the ninth to twelfth day of hormone replacment cycle	EMT	↑	(44)
2016	Prospective study	Infertile women	77	FET	4 IU of rhGH daily from day 3 of the menstrual cycle until the day of progesterone injection	Serum E2 IGF-I VEGF EMT Perfusion of the uterine endometrial arcuate artery IR/CPR/LBR	↑ ↑ ↑ ↑ ↑ ↑	(13)
			76	FET	4 IU of rhGH daily from day 8 of HRT until the day of progesterone injection	EMT IR/CPR	(–) (–)	(13)
2016	Retrospective clinical trial	Infertile women with a normal ovarian response to controlled ovarian hyperstimulation (COH)	556	IVF-ET	4.5 IU of GH daily, starting from the initial day of gonadotropin treatment and lasting for 5 days	EMT in older group (≥35 years old) IR/CPR	↑ ↑	(45)
2018	Randomized controlled trial	Infertile women with RIF	35	IVF/ICSI	NM	EMT IR/CPR	↑ ↑	(12)
2018	Prospective study	Infertile women with RIF	22	IVF-ET	4 IU GH daily until the day of hCG administration	EMT IR/CPR/LBR	↑ ↑	(11)
2018	Randomized controlled trial	Infertile women with thin endometrium (EMT <8 mm)	63	NM	4IU GH daily for 10 days	EMT Uterine perfusion CPR	↑ ↑ ↑	(46)
2018	Prospective study	Infertile women with thin endometrium (EMT <7 mm)	40	IVF/ICSI	5 IU GH daily, starting at the first 4 days till the 18th day of the cycle	EMT IR/CPR	↑ ↑	(14)
2019	Retrospective study	Infertile women with thin endometrium (EMT <8 mm)	184	FET	4.5 IU GH every alternate day, starting from the day of progesterone administration till the day of ET	EMT IR/CPR	(–) ↑	(47)

*↑ = increase; (–) = No significant change*.

*Ref, Reference; NM, Not mentioned*.

*RIF, Repeated implantation failure; IVF, In vitro fertilization; ICSI, Intracytoplasmic sperm injection; FET, frozen-thawed embryo transfer; ET, embryo transfer; rhGH, recombinant human GH; hCG, human chorionic gonadotropin; hMG, human menopausal gonadotropin; EMT, endometrial thickness; FR, Fertilization rate; IR, Implantation rate; CPR, Clinical pregnancy rate; LBR, Live birth rate*.

In patients with repeated implantation failure (RIF), a thicker endometrium on the day of hCG and an increase of IR, CPR and live birth rate (LBR) was found in a GH treated group, consistent with the previous results reported by others (11, 38). In another recent randomized clinical trial (RCT), the patients with RIF in an oocyte donation program also showed an increase of EMT, CPR, and LBR with GH supplements. Since the oocytes were donated by fertile women, further effects of GH on endometrium could be claimed (12).

In infertile women with poor endometrial development (EMT <7 mm), additional GH treatment is alleged to improve the EMT through uterine perfusion as well as the classical endometrial trilaminar pattern, although there was no significant alteration of pregnancy outcomes in this study (40). A meta-analysis including four RCTs demonstrated an enhancement effect of GH on EMT in infertile women with poor endometrial development (EMT <6 mm or non-trilaminar type endometrium) [OR = 10.62, 95% CI (2.97, 38.00)] (48). Other studies also demonstrated that EMT with GH treatment was significantly increased on day 3 (the 18th day of cycle) with subsequent increased IR and CPR (14) and an increased EMT was also detected in five patients with thin endometrium (EMT < 8 mm) after intrauterine perfusion of GH or parenteral injection of GH (41, 44, 46). These observations are not confirmed by others however (13, 37, 39, 47). Different patient selection, doses, starting time as well as the different measurements and interpretations may be the reasons resulting in the varied outcomes of GH treatment (Table 1). This is illustrated in a recent prospective study where the same dose of recombinant human GH (rhGH) was applied to infertile women but with different starting times resulting in quite different clinical outcomes (13). Those women who started with rhGH treatment earlier in the cycle had a significant increase of EMT, perfusion of the uterine artery index, IR, CPR, and LBR as well as estradiol, IGF-I and vascular endothelial growth factor (VEGF) on the day of embryo transfer (13).

## Potential Mechanisms of GH on Endometrial Receptivity

The mechanisms of GH effects on the endometrium to improve the EMT and uterine perfusion and IVF outcomes are still unclear. Currently, several molecules, including IGF, leukemia inhibitory factors (LIF), integrins (Itg), homeobox-containing transcription factors-HOX family genes, etc. contribute to the molecular basis of regulating endometrial receptivity while some molecules closely related to the implantation process have been demonstrated to be involved in the potential mechanisms of GH effects on endometrium (9, 49) (Table 2).

**Table 2:** The effects of GH on endometrial receptivity via molecular biomarkers.

**Years**	**Species**	**Samples**	**Intervention**	**Outcomes**	**Effects**	**References**
**Animal models**						
2005	Porcine	33	6 mg of porcine somatotropin	Endometrial STR, IGF-I, IGF-II, IGFBP2	↑	(25)
2006	Mouse	25	0 15 IU GH/100 g estrous cycle	LIF, Itgαvβ3, MMP-9	↑	(17)
2007	Mouse	25	1.5 m IU GH/g proestrous stage	VEGF, LIF, MMP-9, TIMP-1	↑	(16)
2012	SD rats	25	0 15 IU GH/100 g proestrous stage	Itgαvβ3, OPN	↑	(18)
***In vitro*** **cell studies**						
**Years**	**Cell types**		**Intervention**	**Outcomes**	**Effects**	**References**
2019	RL95-2 cells		Cultured for 48 h in the presence of GH (10 nM)	Cell proliferation; Activates cell cycle; VEGF, ItgB3 and IGF-I	↑	(13)
				Itgαv, LIF, EGF, HOXA10, and SPP1	(–)	(14)
2010	Endometrial stromal cells		4 ng/ml GH; 5 ng/ml IGF-I	Cell proliferation	↑	(15)
	Decidual cells		5 ng/ml IGF-I	Cell proliferation	↑	(15)

*↑ = increase; (-) = No significant change*.

*STR, Somatotropin receptor; IGF(BP), Insulin-like growth factor (binding protein); LIF, Leukemia inhibitory factors; Itg, Integrin; MMP, Matrix metalloproteinase; VEGF, Vascular endothelial growth factor; TIMP, Tissue inhibitor of metalloproteinase; OPN, Osteopontin; SD rats, Sprague Dawley rats; EGF, Epidermal growth factor; HOXA, HOX family; SPP1, Secreted phosphoprotein*.

### Animal Models

Increased concentrations of cytosolic estrogen receptor but not the concentration of progesterone receptor was found in the rabbit uterus after GH treatment, indicating a potential estrogen mediated function (50). Consistent with this, an increase in the concentration of estrogen receptor in the guinea-pig uterus after treating with GH has also been demonstrated (51). Research in the ovine uterus indicates that GH could regulate endometrial gland proliferation via interferon tau (52) and could alter the endometrial gene expression related to maintenance of pregnancy. GH may increase the expression of oxytocin receptor, progesterone receptor mRNA, and the mRNA of estrogen receptor α in non-lactating cows (53) with an increased pregnancy rate in lactating cows after injection of GH at the initiation of timed artificial insemination following a synchronized ovulation protocol (54). Knockout of the GHR in mice leads to a negative impact on reproduction with fewer uterine implantation sites during early pregnancy (55).

GH-mediated increase in uterine IGF-I levels may be the mechanism underlying the increase in endometrial thickness (56). Exogenous porcine GH elevates the expression of endometrial STR, IGF-I mRNA, IGF-II mRNA, and IGFBP2 mRNA in the uterus, supporting the role of the so-called GH/IGF axis in the uterus (25, 56). Both IGF-I and IGF-II can be detected in endometrial stroma, but have different roles (2, 57). IGF-II is more closely related to endometrial differentiation (57), while IGF-I is a potential mediator of the mitogenic effects of estrogen on the uterus, so-called oestromedin (56). Other studies in the mouse also indicate an alteration of other molecular biomarkers of endometrial receptivity after treating GH (16, 17). GH-treated mice show a significant increase of leukemia inhibitory factors (LIF), integrin alpha v beta 3(Itgαvβ3) and matrix metalloproteinase (MMP)-9 in the endometrium, molecules which have been implicated in implantation (16, 17). Exogenous GH supplementation in Sprague Dawley (SD) rats may also increase osteopontin (OPN) and Itgαvβ3 expression with an associated improvement in endometrial receptivity (18).

### *In vitro* Studies

Addition of hGH to the cultured endometrial and decidual cells increases the proliferation of endometrial and decidual cells, when these cells were harvested and separated from the human endometrial pieces and decidual tissue, respectively (15). When transfected to the human endometrial cell line RL95-2, there is an enhancement of cell proliferation, survival and invasion (58) and increased cell proliferation and expression of VEGF, Itgβ3, and IGF-I (14). Janus kinase (JAK) 2 inhibitor AG490 addition with GH suppresses VEGF, Itgβ3, and IGF-I expression in RL95-2 cells, indicating that GH might regulate the expression of these factors via the JAK2 pathway. However, no change in expression of LIF, Itgαv, Hox family gene (HOXA10) and SPP1 could be found in RL95-2 cells after treatment with GH (14). Therefore, the effects of GH on endometrial receptivity-related molecules *in vitro* remains to be elucidated.

## The Potential Risks of GH Treatment in Art

GH may play a pathological role in body systems in view of the fact that it may have a potential of being an oncogene (59). Transplantation of GH transfected-RL95-2 cells suspension to BALBc nu/nu mice led to a larger tumor size and a more aggressive progression of endometrial carcinoma (58). When RL95-2 cells cultured with the GH receptor antagonist (pegvisomant) were inoculated into immunodeficient NIH-III mice and continuously treated the mice with antagonist for 16 weeks, a delayed tumor growth rate and decreased IGF serum level could also be found (60). A systematic review concluded that long-term GH treatment actually had a positive effect on reducing cardiovascular disease, stroke, and fractures, without a simultaneous increase in malignancy risk (61). While there are few indications of problems, the long-term safety of GH for the cancer risk, metabolic disorder and other unforeseen adverse events should be under constant surveillance (62).

Although metabolic sequelae improve with GH in GH deficient patients (63), some advocate that addition of GH in patients with diabetes mellitus should be cautioned due to its potential negative effects on insulin resistance and glucose tolerance (19, 64). GH can also result in significant metabolic changes by elevating cholesterol and disturbing the renin-angiotensin mechanism (65). Therefore, in consideration of the potential risks, personalized comprehensive assessment, and professional guidance in usage and dosage is required before deciding to use GH in ART.

## Conclusion

Clinical evidence for efficacy of GH in improving reproductive function remains controversial. Generally, the majority of studies show positive effects of GH on endometrial receptivity, but there is no general agreement about the dosage and usage of GH in ART and there are few useful RCTs (Table 1). Hence, more evidence is still required to determine the purported value of GH treatment in ART and provide more specific guidance in the clinical setting.

Even if clinical evidence currently encourages the view that GH might be helpful for endometrial receptivity, the mechanisms are still not known. The studies of molecular biomarkers included in this review are few (Table 2), but also may provide some foundations for future exploration of the mechanisms of GH in the endometrium. As GH is administered systemically during ART, it is difficult to separate the effect on GHR in the ovary from that in the endometrium and if GH alters receptor action in the ovary, it could potentially have an effect on the endometrium receptors too.

The risk of GH used in ART should be noticed but not overstated. There is a relationship between autocrine GH and endometrial cancer, but further studies of the mechanisms under this phenomenon and the confirmation of increased diseases risk of exogenous GH are still needed. In general, further explorations of the mechanisms underlying the effects of GH on endometrium should spread some light on our basic knowledge and clinical actions.

## Author Contributions

F-TL and RL were responsible for writing the first draft. RL, ZW, JY, and RN performed the critical revisions. All authors listed did contributed to the writing and review of the manuscript.

### Conflict of Interest

The authors declare that the research was conducted in the absence of any commercial or financial relationships that could be construed as a potential conflict of interest.
